# Management of Children With Fever at Risk for Pediatric Sepsis: A Prospective Study in Pediatric Emergency Care

**DOI:** 10.3389/fped.2020.548154

**Published:** 2020-09-17

**Authors:** Ruud G. Nijman, Rikke Jorgensen, Michael Levin, Jethro Herberg, Ian K. Maconochie

**Affiliations:** ^1^Department of Paediatric Accident and Emergency, St. Mary's Hospital – Imperial College NHS Healthcare Trust, London, United Kingdom; ^2^Section of Paediatric Infectious Diseases, Department of Infectious Diseases, Faculty of Medicine, Imperial College London, London, United Kingdom

**Keywords:** child, fever, sepsis, pediatric sepsis interventions, clinical tools

## Abstract

**Objective:** To study warning signs of serious infections in febrile children presenting to PED, ascertain their risk of having sepsis, and evaluate their management.

**Design:** Prospective observational study.

**Setting:** A single pediatric emergency department (PED).

**Participants:** Febrile children, aged 1 month−16 years, with >= 1 warning signs of sepsis.

**Interventions and Main outcome measures:** Clinical characteristics, including different thresholds for tachycardia and tachypnoea, and their association with (1) delivery of pediatric sepsis 6 (PS6) interventions, (2) final diagnosis of invasive bacterial infection (IBI), (3) the risk for pediatric intensive care unit (PICU) admission, and (4) death.

**Results:** Forty-one percent of 5,156 febrile children had warning signs of sepsis. 1,606 (34%) children had tachypnoea and 1,907 (39%) children had tachycardia when using APLS threshold values. Using the NICE sepsis guidelines thresholds resulted in 1,512 (32%) children having tachypnoea (kappa 0.56) and 2,769 (57%) children having tachycardia (kappa 0.66). Of 1,628 PED visits spanning 1,551 disease episodes, six children (0.4%) had IBI, with one death (0.06%), corresponding with 256 children requiring escalation of care according to sepsis guideline recommendations for each child with IBI. There were five additional PICU admissions (0.4%). 121 (7%) had intravenous antibiotics in PED; 39 children (2%) had an intravenous fluid bolus, inotrope drugs were started in one child. 440 children (27%) were reviewed by a senior clinician. In 4/11 children with IBI or PICU admission or death, PS6 interventions were delivered within 60 min after arriving. 1,062 (65%) visits had no PS6 interventions. Diagnostic performance of vital signs or sepsis criteria for predicting serious illness yielded a large proportion of false positives. Lactataemia was not associated with giving iv fluid boluses (*p* = 0.19) or presence of serious bacterial infections (*p* = 0.128).

**Conclusion:** Many febrile children (41%) present with warning signs for sepsis, with only few of them undergoing investigations or treatment for true sepsis. Children with positive isolates in blood or CSF culture presented in a heterogeneous manner, with varying levels of urgency and severity of illness. Delivery of sepsis care can be improved in only a minority of children with IBI or admitted to PICU.

## Introduction

A majority of children with an acute infectious illness presenting to emergency care facilities will have a self-limiting illness (1). Still, an estimated 1,000 children with sepsis are being admitted to pediatric intensive care units in the United Kingdom annually, and sepsis accounts for more than 10% of childhood deaths in children aged <4 years (2). Typically, many children with sepsis will present with non-specific signs and symptoms early in their disease course, with the full severity of the illness becoming manifest only later (3, 4).

Worse outcomes in children with sepsis presenting to emergency departments are associated with the failure to recognize children with sepsis early, the absence of specialist supervision, and subsequent failure of escalating care to a more senior clinician (4), as well as delayed administration of parenteral antibiotics (5). Thus, clinical tools or decision support algorithms for aiding physicians in their decision making could benefit children with possible sepsis (6, 7).

Sepsis is traditionally defined by criteria such as those of the Systemic Inflammatory Response Syndrome (SIRS) or Sequential Organ Assessment Failure (SOFA) criteria (8–11), and typically include some clinical signs as well biochemical evidence of end-organ impairment. However, making a clinical diagnosis of sepsis continues to depend on recognizing clinical signs and symptoms. Most clinical algorithms are based on abnormal vital parameters, such as heart rate, respiratory rate, temperature, capillary refill time, and decreased level of consciousness. However, these abnormal vital parameters are also seen in a large proportion of children whose fever is due to self-limiting infectious disease (12). In addition, children are often able to maintain normal haemodynamic parameters in the early stages of sepsis, complicating the use of vital sign-based tools for the detection of sepsis.

The National Institute of Health Care Excellence (NICE) in the United Kingdom published guidelines to identify children at increased risk for sepsis in 2016 (13). The guideline recommendations specifically focus on the importance of early escalation of care in children at increased risk of sepsis, and on expediting clinical interventions, such as intravenous antibiotics and intravenous fluid bolus.

Understanding the routine management of children at increased risk for sepsis in emergency care will be important in estimating the potential impact of guidance advocating the early detection and management of sepsis. This prospective observational study aimed to study warning signs of serious infections in febrile children presenting to PED, ascertain their risk of having sepsis, and evaluate their management.

## Methods

In this prospective observational study, we first described the characteristics of children with fever presenting to PED and their clinical outcomes. We then assessed the value of heart rate and respiratory rate for identifying children at risk for sepsis. Finally, we evaluated the clinical management of children with warning signs of sepsis, with a specific focus on those children admitted to PICU and a confirmed invasive bacterial infection.

### Design, Setting, and Participants

This prospective observational study (Infections in Children in the Emergency Department (ICED)—study) was conducted at the PED at St. Mary's Hospital, Imperial College NHS Healthcare Trust, London, United Kingdom. The PED in this large teaching hospital in central London sees about 27,000 children a year, set in a hospital that is a tertiary referral center for pediatric hematology and infectious diseases, is a major trauma center and has a 10 bedded pediatric intensive care unit. The PED is staffed with resident medical officers; patients seen by junior trainees below registrar level are supervised by a senior registrar who completed a minimum of 3 years pediatrics or emergency medicine specialty training (ST3+ level). There is a supervising consultant with pediatric emergency specialization available daily on week days between 8 a.m. and 8 p.m., being non-resident on call at night the remaining hours. At weekends, there is a PED consultant resident for 3 h a day, the rest of the time being covered on call out of the department. A senior pediatric registrar is available to review PED patients 24 h a day and every day of the week. The nursing staff all specialize in pediatric emergency care, with initial triage being performed by a senior staff nurse from this team.

Prospective data were collected for all children aged 1 month to 16 years old presenting with fever, for the period of June 2014–March 2015. Fever was defined:

1. A tympanic or axillary fever ≥38.0 degrees Celsius measured at triage,
2. “Fever” as discriminator in the Manchester Triage System (14),
3. Fever as the reason for GP referral to PED,
4. Fever above 38 degrees Celsius measured at home in the 24 h prior to presentation.

Additional in-depth data on subsequent diagnostic investigations, clinical interventions, and final outcomes were coded for febrile children at increased risk of sepsis, defined as fever in the presence of one or more red or ambers signs as defined by the NICE guideline for the management of fever as identified by the triaging nurse (15). Clinical interventions of interest included those as described in the pediatric sepsis six (PS6) care bundle performed in the PED (13, 16) as per Table 1.

**Table 1:** Clinical interventions as defined by the pediatric sepsis six care bundle.

**• Senior review, defined as a specialty trainee in pediatrics or emergency medicine with a minimum of 3 years specialty experience (ST3+) or above**
**• Sampling of blood tests, blood gas and/or blood cultures**
**• Administration of intravenous and/or intramuscular antibiotics**
**• Intravenous fluid bolus^∧^**
**• Oxygen via non-invasive ventilation, i.e., facial oxygen or non-rebreathing mask, nasal cannula with low flow, or high flow oxygen**
**• Inotropic support, either peripherally, or centrally administered**

∧ *Not intravenous maintenance fluids*.

Additionally, we reviewed the records of any child presenting to PED that was admitted to PICU or died, but whom presented without fever or was not eligible for inclusion in the final ICED cohort. Children with a complex medical history, defined as an underlying medical problem requiring >=2 annual visits to a pediatric specialist over a period >12 months, were excluded from the final cohort (17). Similarly, we excluded (1) children who did not wait to be seen, (2) children who were discharged to urgent care center without being seen in PED, (3) non-UK citizens, (4) children when all clinical data of the visit was missing, and (5) if the physician overruled the presenting problem of fever or the warning signs assigned to the patient at triage and actively deemed the patient not eligible for the study.

A waiver of patient informed consent was obtained from the local medical ethical committee as only routine clinical data were used (14/LO/0266). However, a biomarker discovery study was embedded into this study and for that study patient informed consent was required; hence, we excluded a small number of children from our cohort that did not consent to participation in the biomarker discovery study.

### Outcome Measures

As a primary outcome, we determined the incidence of febrile children presenting to PED with warning signs of sepsis, how often these children fulfilled pediatric sepsis criteria, and how frequent invasive bacterial infections and PICU admissions occurred in this population. As a secondary outcome measure, we studied the expedience measures and resource utilization of children at risk of sepsis and the compliance with the proposed course of management according to the pediatric sepsis 6 care bundle (Table 1). We calculated expediency measures and resource utilization for each unique PED visit; we coded a single final diagnosis and the need for hospital admission for each disease episode, accounting for return visits with a similar illness within a five-day period.

#### Working Diagnosis and Diagnosis of Serious Bacterial Infection

A working diagnosis was determined by the treating physician and electronically coded at the time of discharge from the PED. In addition, a coded final diagnosis of serious bacterial infection (SBI) was based on a previously published reference standard, which included positive cultures from sterile sites [blood, urine, cerebrospinal fluid (CSF)], additional microbiology, virology and radiology data, and consensus diagnosis by members of the clinical research team (RN, RJ, JH) (18, 19). A diagnosis of bacterial pneumonia was based on radiographic evidence of consolidation or effusion as determined by a pediatric radiologist. Similarly, invasive bacterial infections (IBI) were defined as those with positive bacterial isolates in blood or CSF. The research team coded a single final outcome diagnosis for every disease episode, considering data from all PED visits within a 5-day window and, if admitted, data from the complete hospital admission duration.

#### Pediatric Sepsis Criteria and Different Thresholds of Vital Signs

We looked at the number of children having tachycardia or tachypnoea by using different thresholds as defined by (1) Advanced Pediatric Life Support (APLS) thresholds and (2) thresholds used in the NICE guidelines on the early detection and management of sepsis (13), which correspond with the 99th centiles for respiratory rate and heart rate described in the meta-analysis by Fleming et al. (20). We then looked at the number of children fulfilling sepsis criteria using several sepsis scores, namely (1) amber or red signs as described in the NICE guidelines on the early detection and management of sepsis; (2) the age adjusted SIRS and qSOFA scores as defined according to Goldstein et al. (10) and Schlapbach et al. (21) (Appendix A); (3) the Sepsis Trust UK trigger criteria as defined by >=2 of: temperature >38.5 degrees Celsius, inappropriate tachycardia (APLS thresholds), altered mental state, and prolonged capillary refill (22).

#### Expediency Measures and Resource Utilization

Expediency measures were the timeliness of interventions being undertaken, broken down into the following categories about the time to achieve the following: time to senior review, time to intravenous antibiotics, time to intravenous access and blood sampling, and time to inotropic support; these were measured in minutes from time of arrival to time of intervention. Resource utilization was based on the items of the PS6 care bundle (Table 1).

### Data Collection

Standardized, electronic data on triage, vital signs, clinical signs and symptoms, diagnostic tests, working diagnosis and need for hospital admission were recorded prospectively for all febrile children in their digital file as part of routine clinical care. These data were collected using a predefined data collection form integrated into the electronic file of the patient using SYMPHONY software (23). For febrile children with one or more of the above warning signs, additional data were entered in an electronic data entry form by the research team, including data on time to interventions, which were based on real-time data registration in the patient's electronic file or written medical notes. All medical and nursing staff had undergone training in advanced life support for children, having attended relevant certified life support courses.

### Statistical Analysis

Chi square analyses were used for categorical and dichotomous variables, and Fisher's exact test was used when <=5 cases present; non-parametric tests of Mann Whitney-U or Kruskall-Wallis were used for non-normally distributed continuous variables, and student's *t*-test was used for normally distributed continuous variables. Cohen's kappas were calculated for the level of agreement between definitions of abnormal heart rate and respiratory rate as defined by APLS and the NICE sepsis guidelines (either RED or AMBER). Diagnostic performance of the different thresholds of vital signs, sepsis criteria, and lactate to predict presence of SBI, IBI and PICU admission were calculated. In line with our study objectives, we used available data only without imputing missing data. SPSS version 25 IBM, Chicago Inc., and R statistical software, version 3.6.1, were used for statistical analyses.

## Results

### Study Population

**Figure 1:**
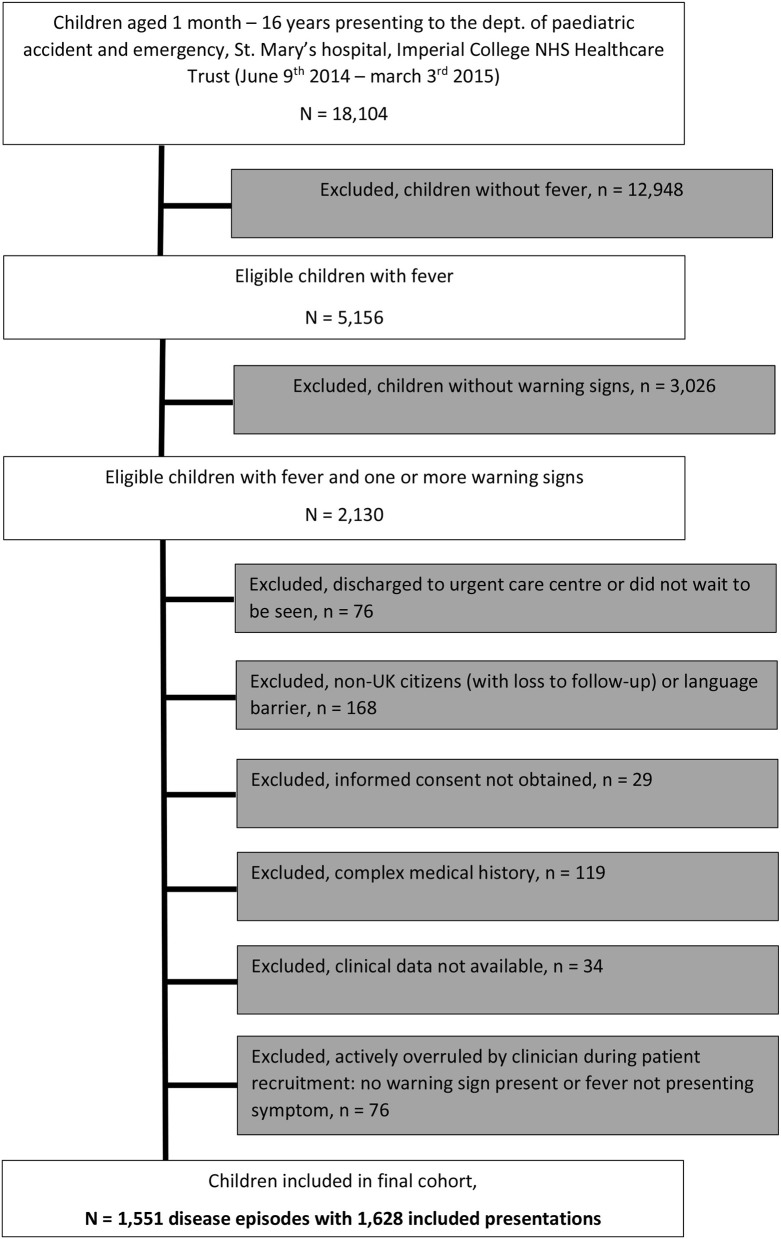
Flowchart of included population.

Of 18,104 children aged 1 month to 16 years presenting to PED, 5,156 had fever (28%). Of these, 2,130 (41%) had one or more warning signs. A total of 1,628 unique visits for fever belonging to 1,551 disease episodes (considering all visits of an individual patient within a 5-day period) were ultimately included in the final cohort of febrile children with warning signs (Figure 1). In total, 255/1,551 (16%) children in this cohort had more than one visit to PED for any problem during a disease episode; of these children, a majority had two visits during the disease episode (218/255 children (85%), range 2–5 visits); 1,458/1,551 (94%) children were included during their first presentation to ED; the remaining 93/1,551 (6%) children had presented to ED previously without fever and/or warning signs. In total, 228/1,551 (15%) children were admitted following their first presentation to ED; a further 38/1,551 (2%) children were (re)admitted to hospital after a revisit, and 12 of these children (0.7%) had SBI. Overall, hundred and eleven children had an SBI (7%); 266/1,551 children (17%) were admitted, and 716/1,551 children (46%) were given any type of antibiotics at any stage during the disease episode (Tables 2, 3).

**Table 2:** Description of study population.

**General characteristics**		**All children with fever**	**Included cohort of children with fever and **>=** 1 risk factor of sepsis**
		**5,156 PED visits**	**1,551 disease episodes with 1,628 PED visits**
Age	Years (median, IQR)	2.99 (1.40–5.69)	2.58 (1.40–5.24)
	1 month– <1 year (*n*, %)	809 (16%)	219 (14%)
	1– <2 years (*n*, %)	1,062 (21%)	387 (25%)
	2– <5 years (*n*, %)	1,754 (34%)	528 (34%)
	5– <16 years (*n*, %)	1,531 (30%)	417 (27%)
Gender	Male (*n*, %)	2,795 (54%)	855 (55%)
Time of the day	07.00–16.00 h (*n*, %)	1,821 (35%)	573 (35%)
	16.00–23.00 h (*n*, %)	2,428 (47%)	741 (46%)
	23.00–07.00 h (*n*, %)	907 (18%)	314 (19%)
Triage urgency^a^	Emergent (to be seen immediately) (*n*, %)	43 (0.8%)	20 (1.2%)
	Very urgent-(within 10 min) (*n*, %)	1,362 (26%)	789 (49%)
	Urgent (within 60 min) (*n*, %)	867 (17%)	279 (17%)
	Standard (within 120 min) (*n*, %)	2,860 (56%)	538 (33%)
	Non-urgent (within 240 min) (*n*, %)	24 (0.5%)	2 (0.2%)
Heart rate	Tachycardia [APLS thresholds, *n* (%)]	1,907 (39%)^b^	1,184 (73%)
	Missing values (*n*, %)	277 (5%)	52 (3%)
Respiratory rate	Tachypnoea [APLS thresholds, *n* (%)]	1,606 (34%)^b^	850 (52%)
	Missing values (*n*, %)	426 (8%)	88 (5%)
Oxygen saturations	In %O2 (median, IQR)	99 (98–100)	99 (97–100)
	<94% O2 (*n*, %)	99 (2%)^b^	52 (3%)
	Missing values (*n*, %)	277 (5%)	55 (3%)
Body temperature	Degrees Celsius (median, IQR)	37.8 (37.0–38.4)	38.5 (38.0–39.0)
	Missing values (*n*, %)	354 (7%)	71 (4%)
Capillary refill	<= 2 s (*n*, %)	3,659 (99%)	1,268 (98%)
	>2– <4 s (*n*, %)	41 (1.1%)^b^	26 (1.6%)
	>=4 s (*n*, %)	4 (0.1%)^b^	2 (0.1%)
	Missing values (*n*, %)	1,452 (28%)	332 (20%)
AVPU	Alert (*n*, %)	4,798 (100%)	1,539 (99%)
	Voice (*n*, %)	15 (0.3%)^b^	8 (0.5%)
	Pain (*n*, %)	2 (<0.1%)^b^	2 (0.1%)
	Unresponsive (*n*, %)	3 (0.1%)^b^	1 (0.1%)
	Missing values (*n*, %)	338 (7%)	78 (5%)
Blood pressure	Measured in *n* (%) of patients	396 (8%)	120 (7%)
Lactate	Number of patients (%) with available value	n/a	149 (9%)
	mMol/L (median, IQR)	n/a	1.7 (1.2–2.5)
C-reactive protein	Number of patients (%) with available value	n/a	291 (18%)
	mg/L (median, IQR)	n/a	24 (8–63)

a *The Manchester triage System was used to designate a level of urgency of care (14)*.

b *Several children with O2 saturations <94% were not included in final cohort, as they had a history of fever, but not a measured temperature in PED, and were managed for a respiratory wheeze episode rather than an infectious febrile episode. Similarly, some children with tachycardia, tachypnoea, prolonged capillary refill, and some children with V/P/U on AVPU scale were excluded due to an absence of fever in PED and management for other presenting problems*.

*n/a not available*.

**Table 3:** Outcomes in study population.

**Outcomes**		**All children with fever**	**Included cohort of children with fever and **>=** 1 risk factor of sepsis**
		**5,156 PED visits**	**1,551 disease episodes with 1,628 PED visits**
Admission	Pediatric ward (*n*, %)	580 (11%)	266 (17%)
	PICU (*n*, %)	8 (0.2%)^a^	5 (0.4%)
	Deceased in emergency dept (*n*, %)	1 (0.1%)	1 (0.1%)
Antibiotics	Any type of antibiotics given at any time in this febrile disease episode (*n*, %)	-	716 (46%)
	Parenteral (IM/IV)^b^ (*n*, %)	-	165 (11%)
Serious bacterial infection	Meningitis, Bacteraemia or sepsis (*n*, %)	-	6 (0.4%)
	Urinary tract infection (*n*, %)	-	23 (1.5%)
	Pneumonia (*n*, %)	-	43 (2.8%)
	Other SBI (*n*, %)^c^	-	39 (2.5%)
	Non SBI (*n*, %)	-	1,440 (93%)

a An additional three children admitted to PICU (Appendix D) had fever and warning signs, but were excluded from final analysis as described in “Management of children admitted to PICU.”

b *Parenteral antibiotics given IV or IM in PED or on the ward following admission to hospital*.

c *Other SBI include bacterial gastro-enteritis (n = 5), abscess (n = 12), serious focal skin infection (n = 7), pre-septal or orbital cellulitis (n = 7), mastoiditis (n = 1), appendicitis (n = 4), peritonitis and hypovolaemic shock (n = 1), scarlet fever (n = 1), and aseptic, culture negative, meningitis with pre-treatment (n = 1)*.

*IM, intramuscular; IV, intravenous; PED, pediatric emergency department; PICU, pediatric intensive care unit*.

### Vital Signs and Sepsis Criteria

**Figure 2:**
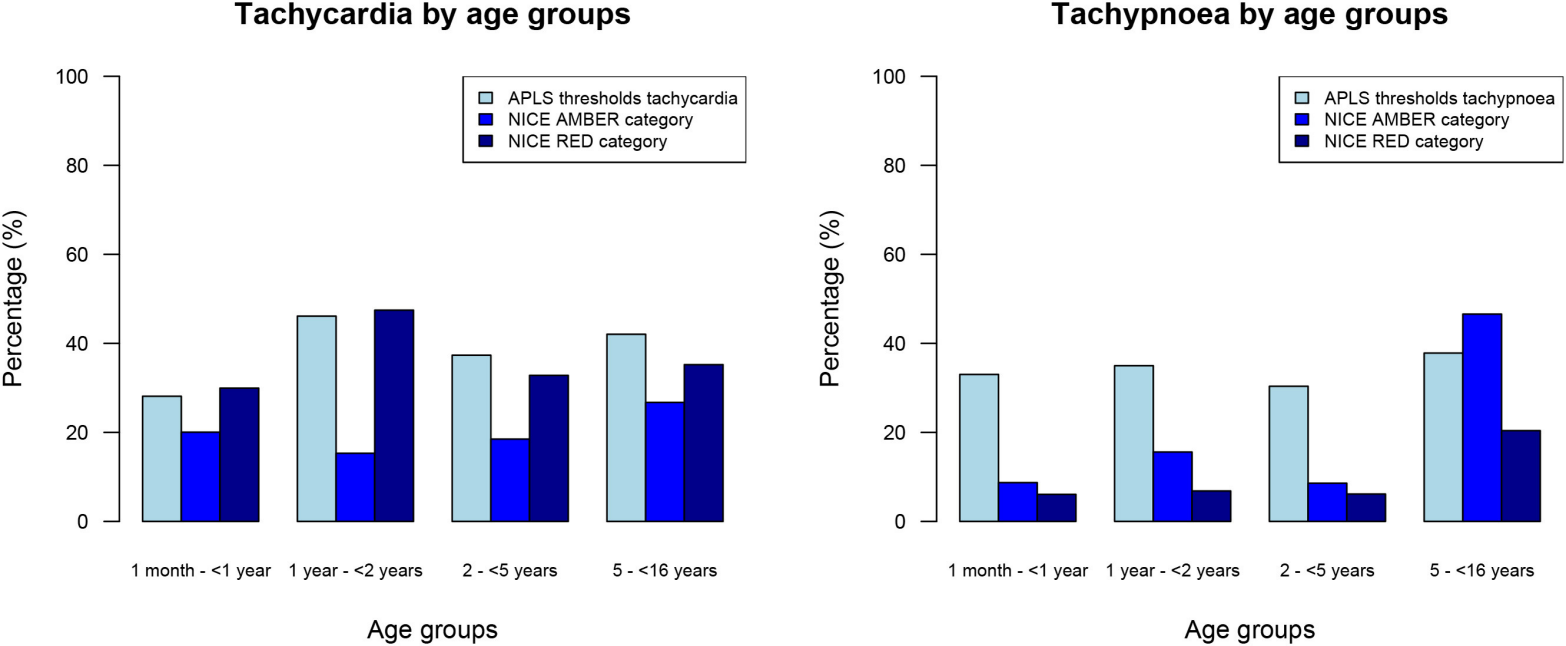
Percentages of all febrile children with tachycardia and tachypnoea, categorized by age groups.

In our population of all febrile children, 1,606/4,730 (34%) children had tachypnoea and 1,907/4,897 (39%) children had tachycardia when using APLS threshold values (Table 2). Using the NICE sepsis guidelines thresholds resulted in 1,512/4,730 (32%) children having tachypnoea and 2,769 (57%) children having tachycardia (Figure 2, Appendix B). This reclassification meant that a higher proportion of children aged >5 years had AMBER or RED signs for tachypnoea, with fewer children aged under 5 years classifying as AMBER or RED; for tachycardia, children were classified more often as AMBER or RED across all ages, mostly because of lower thresholds to define tachycardia in the AMBER category. For respiratory rate, Cohen's kappa to define tachypnoea was 0.56 (“weak” level of agreement, standard error 0.01, *p*-value < 0.001) between APLS thresholds and NICE sepsis (RED or AMBER) thresholds; for tachycardia this was 0.66 (“moderate” level of agreement, standard error 0.01, *p*-value < 0.001).

For the cohort of children with warning signs, 1,441/1,628 (89%) had one or more abnormal vital signs (Table 4). Of those children with warning signs and with normal vital signs, “decreased activity” (Amber sign, *n* = 16/187, 9%), “fever for more than or equal to 5 days” (Amber sign, *n* = 30/187, 16%), and “no smile” (Amber sign, *n* = 42/187, 22%) were the most frequent. A total of 545 (33%) children had >=2 positive SIRS items, and 1,317 children (81%) had one or more positive qSOFA items. A substantial number of children did not have data available for all required parameters, mostly owing to non-availability of systolic blood pressure, which was measured only in 120 children (7%), and white cell count, which was measured in 293 children (18%). Tachycardia was present in all 10 children with IBI or PICU admission; noticeable was the high rate of missing blood pressures as part of initial observations in this group of patients (Table 5). Overall, diagnostic performance of vital signs and sepsis criteria were insufficient to predict presence of SBI or IBI/PICU, with a large proportion of false positives (Appendix C).

**Table 4:** Pediatric sepsis criteria and sepsis warning scores.

		***N (%)***
**Advanced pediatric life support thresholds (APLS):**		
Abnormal vital signs	Fever and any abnormal vital sign	1441/1628 (89%)
	Tachycardia	1184/1576 (75%)
	Tachypnoea	850/1540 (55%)
**Categories of NICE management of feverish illness traffic light system^a^**		
AMBER (fever)	Any positive symptom or sign	726/1628 (45%)
RED (fever)	Any positive symptom or sign	51/1628 (3%)
**NICE sepsis guideline: thresholds for vital signs**		
	Tachycardia AMBER	285/1576 (18%)
	**Tachycardia RED**	**1072/1576 (68%)**
	Tachypnoea AMBER	407/1540 (26%)
	**Tachypnoea RED**	**259/1540 (17%)**
**Systemic Inflammatory Response Syndrome (SIRS)^b^**		
	1 item positive	561 (34%)
	**2 items positive**	**545 (33%)**
	3 items positive	329 (20%)
	4 items positive	26 (2%)
	All parameters available	268 (17%)
**Quick Sequential Organ Assessment Failure (qSOFA)^b^**		
	1 item positive	1313 (81%)
	**2 items positive**	**8 (0.5%)**
	3 items positive	0
	All parameters available	112 (7%)
**Sepsis Trust criteria^b^**		
	1 item positive	714 (44%)
	**2 items positive**	**563 (35%)**
	3 items positive	24 (1%)
	4 items positive	1 (0.1%)
	All parameters available	1178 (72%)

a *Categories of NICE management of feverish illness traffic light system (15): as identified by nurse at the time of triage by using the NICE traffic light system. For SIRS, qSOFA, and Sepsis Trust criteria, the positive items were derived from the vital signs as measured at time of triage. Note high frequencies of missing data for SIRS (WBC present in 16%) and qSOFA (BP present in 7%)*.

b *Appendix A for definitions of SIRS, qSOFA and Sepsis Trust; any one NICE RED or any two NICE AMBER should trigger escalation of care and senior clinician review; SIRS, qSOFA and Sepsis Trust criteria considered positive if 2 or more items present [in bold]. For 1,628 PED visits*.

**Table 5:** Febrile children with invasive bacterial infection or admitted to PICU and sepsis scores.

	**Age**	**Gender**	**Vital signs^a^**	**Outcome**	**IBI**	**Vitals signs and sepsis scores^b^**							
						**APLS tachycardia**	**APLS tachypnoea**	**NICE tachycardia^c^**	**NICE tachypnoea^c^**	**NICE fever triage code^d^**	**qSOFA**	**SIRS**	**Sepsis trust**
1	4 years	Male	BP missing	Admitted	YES	No	Yes	AMBER	AMBER	GREEN	Negative (score:1)	Positive (score:2)	Negative (score: 0)
2	11 years	Female	BP missing	Admitted	YES	Yes	No	RED	AMBER	*Missing*	Negative (score:1)	Positive (score:4)	Positive (score:2)
3	3 years	Female	BP missing	Admitted	YES	Yes	No	RED	GREEN	AMBER	Negative (score:1)	Positive (score:3)	Positive (score:2)
4	2 months	Male	BP missing	Admitted	YES	Yes	Yes	RED	AMBER	*Missing*	Negative (score:1)	Negative (score:1)	Negative (score:1)
5	8 months	Female	RR/BP missing	Admitted	YES	Yes	*Missing*	RED	*missing*	AMBER	Negative (score: 0)	Positive (score:2)	Negative (score:1)
6	11 years	Female	BP/CR missing	PICU	NO	Yes	Yes	RED	RED	GREEN	Positive (score:2)	Positive (score:3)	Positive (score:3)
7	2 years	Female	Complete	PICU	NO	Yes	No	RED	GREEN	AMBER	Negative (score:1)	Positive (score:3)	Negative (score:1)
8	1 month	Male	BP/CR missing	PICU	NO	Yes	Yes	RED	RED	AMBER	Positive (score:2)	Negative (score:1)	Positive (score:2)
9	1 years	Female	BP missing	PICU	NO	Yes	No	RED	GREEN	*Missing*	Negative (score:1)	Positive (score:2)	Positive (score:4)
10	10 years	Male	CR missing	PICU	NO	Yes	No	RED	AMBER	AMBER	Negative (score:1)	Positive (score:4)	Positive (score:2)
11	1 years	Male	In arrest	RIP	YES								
Total positive:						9/10 (90%)	4/9 (44%)	10/10 (100%)	6/9 (67%)	5/7 (71%)	2/10 (20%)	8/10 (80%)	6/10 (60%)

a *Vital signs include: HR, RR, BP, CR, AVPU score, temperature, O2 saturations*.

b *Appendix A for definitions of SIRS, qSOFA and Sepsis Trust; any one NICE RED or any two NICE AMBER should trigger escalation of care and senior clinician review; SIRS, qSOFA and Sepsis Trust criteria considered positive if 2 or more items present*.

c *Adjusted for value measured at time of triage using the NICE sepsis guidelines thresholds for tachycardia and tachypnoea*.

d *As completed by triaging nurse*.

*BP, blood pressure; CR, capillary refill; HR, heart rate; IBI, invasive bacterial infection; PICU, pediatric intensive care unit; RR, respiratory rate*.

*The green coloured boxes highlight those cases that score positive*.

### Management of Children at Risk of Serious Infections

A minority of febrile children with warning signs received any of the PS6 interventions in the PED (Table 6). Expediency of pediatric sepsis 6 interventions varied widely, with five children receiving all PS6 interventions in the first hour after arriving; one of those had inotrope drugs commenced after 60 min in PED. Thousand and sixty-two (65%) children had none of the PS6 interventions.

**Table 6:** Pediatric Sepsis 6 interventions and timeliness of interventions in the PED.

**Pediatric sepsis 6 (PS6) interventions in PED**			
		**All children with fever and warning signs^a^**	**Children with IBI/PICU** ***N* = 11**
No PS6 interventions	N (%)	1,062 (65%)	0
Intravenous access	N (%)	265 (16%)	11 (100%)
Blood gas	N (%)	152 (9%)	10 (91%)
	Within 60 min of ED arrival	24 (1%)	6 (55%)
	Time to (median, IQR)	119 (84–166)	55 (41–148)
Intravenous antibiotics	*N* (%)	121 (7%)	10 (91%)^c^
	Within 60 min of ED arrival	12 (0.7%)	5 (45%)
	Time to (median, IQR)	184 (114–235)	40 (23–197)
Intravenous fluid bolus	*N* (%)	39 (2%)	6 (55%)
	Within 60 min of ED arrival	8 (0.5%)	4 (36%)
	Time to (median, IQR)	114 (61–172)	49 (25–154)
Senior review by doctor of ST3 grade and above	*N* (%)	440 (27%)	11 (100%)
	Within 60 min of ED arrival	114 (7%)	5 (45%)
	Time to senior review (median, IQR)	99 (52–161)	121 (39–217)
Inotrope drugs	*N* (%)	1 (0.1%)	1 (9%)
	Within 60 min of ED arrival	0	0
Golden hour: all PS6 interventions within first hour after arriving^b^	*N* (%)	5 (0.3%)	4 (36%)

a *Based on number of visits, n = 1,628*.

b *Obtaining iv access; performing blood culture and additional laboratory investigations; performing blood gas and lactate; administering iv fluid bolus and iv antibiotics; senior review; excluding initiating inotrope drugs within the first 60 min after arriving*.

c *1 child received antibiotics on admission to the ward and not in PED*.

*PED, pediatric emergency department; IBI, invasive bacterial infection; PICU, pediatric intensive care unit*.

**Figure 3:**
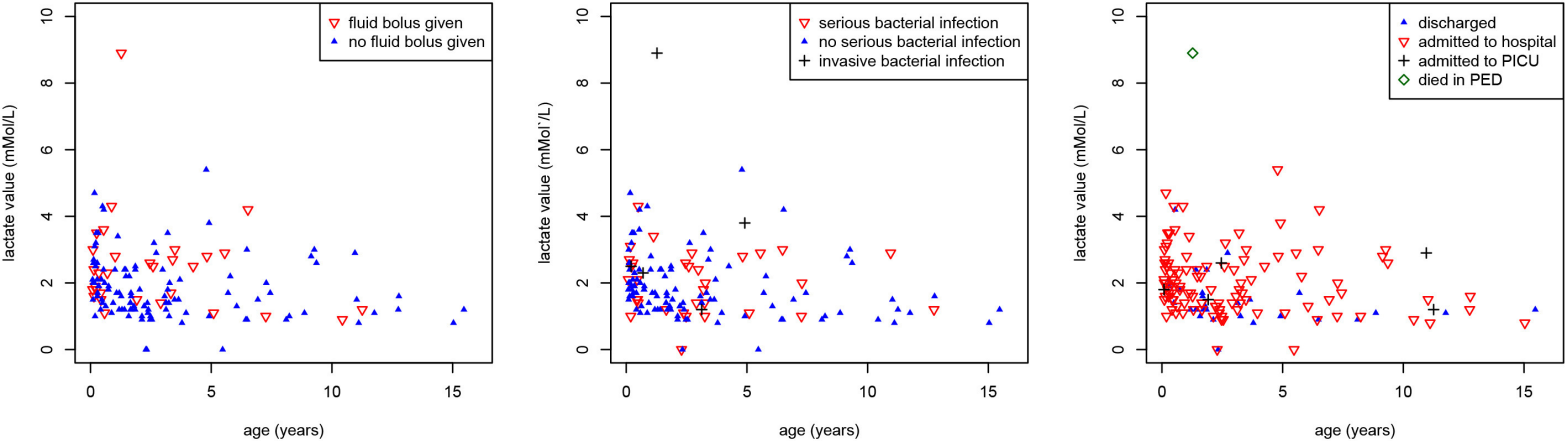
Associations between lactate values, age and outcomes. Scatter plot showing the associations between lactate values (mMol/L) (y-axis), age (years) (x-axis) and (a) intravenous fluid bolus (red inverse triangles) (*p* = 0.19) [left], (b) serious (red inverse triangles) and invasive (black plus sign) bacterial infections (*p* = 0.128) [middle], (c) hospital admission (red inverse triangles), PICU admission (black plus sign), and death (green diamond) (*p* = 0.002) [right].

Eighty-six of 149 (58%) children had lactate <2 mMol/L, 56 (38%) between 2 and 4 mMol/L, and 7 (5%) had a lactate level >= 4 mMol/L. This was not related to the decision of giving an IV fluid bolus (*p* = 0.19) or the presence of serious bacterial infections (*p* = 0.128) (Figure 3). There was a significant association between lactate levels and hospital admission (*p* = 0.002). A lactate level of >2 mMol/L had a sensitivity of 0.60 (95% CI 0.27–0.86), specificity of 0.64 (95% CI 0.55–0.72), positive LR 1.67 (95% CI 0.96–2.90), and negative LR 0.62 (95% 0.29–1.35) for IBI or PICU admission. For SBI, this was a sensitivity of 0.53 (95% CI 0.36–0.68), specificity of 0.68 (95% CI 0.58–0.76), positive LR 1.64 (95% CI 1.09–2.44), and negative LR 0.70 (95% 0.50–0.98). A high percentage (79%) of children who had a lactate value measured were admitted to hospital compared with the overall rate of admission (17%).

### Management of Children Admitted to PICU

Five children (0.3%) were admitted to PICU whilst one child (0.06%) died in ED (Tables 7a,b). One child was admitted to PICU with bronchiolitis after having been discharged from the pediatric ward the previous day (Table 8). One child admitted to PICU with intussusception had presented twice previously with vomiting, some 21 and 32 h beforehand, respectively. One child died of sepsis in the ED; this child had presented 8 h beforehand with fever and upper respiratory tract focus, and was discharged home following normalization of all vital signs during the first visit. This child received all the pediatric sepsis 6 interventions within 1 h of arriving on the second and final attendance. For the children admitted to PICU most PS6 interventions were delivered in the ED, but the time to the delivery of the PS6 interventions varied (Tables 6, 7a).

**Table 7a:** Management of children admitted to PICU (*n* = 5).

	**Age**	**Gender**	**Triage code^a^**	**Vital signs**	**Final diagnosis**	**IBI**	**CRP^b^ (mg/L)**	**PS6 interventions in the emergency department (time to intervention)**						
								**Iv access**	**Blood lactate (mMol/L)**	**Blood culture**	**Iv antibiotics**	**Iv fluid bolus**	**Senior review**	**Inotropes**
1	11 years	Female	Unwell child, Hot child, very urgent	HR 136; RR 32; Temp 40.5; sats 98%; AVPU voice	Encephalitis Npa: influenza A virus Clinical deterioration in PED	No	21	Yes (24 min)	Yes 1.2 mMol/L (42 min)	Yes	Yes (189 min)	Yes (time unknown)	Yes (274 min)	No
2	1 years	Female	Fits, Airway compromised, emergent	HR 185; RR 30; T 39; CR >2 - <4 s; sats 100%; AVPU unresponsive	Status Epilepticus Npa: RSV and influenza A	No	5	Yes (8 min)	Yes 1.5 mMol/L (55 min)	Yes	Yes (8 min)	Yes (8 min)	Yes (<10 min)	No
3^*^	2 years	Female	Abdominal Pain in Children, Signs of severe pain, very urgent	HR 160; RR26; T 37.9; CR 2 s or less; BP 94/63; sats 100%; AVPU alert	Intussusception PICU after theaters	No	332	Yes (31 min)	Yes 2.6 mMol/L (31 min)	Yes	Yes (38 min)	Yes (38 min)	Yes (28 min)	No
4	1 month	Male	Shortness of breath in children, Increased work of breathing, very urgent	HR 170; RR 110; T38.3; sats 88%; AVPU pain	LRTI Npa: human metapneumovirus Initially optiflow then intubated in PED	No	59	Yes (9 min)	Yes 1.8 mMol/L (11 min)	Yes	Yes (15 min)	Yes (60 min)	Yes (<10 min)	No
5	10 years	Male	Abdominal Pain in Children, Signs of moderate pain, urgent	HR 160; RR 22; T 40; BP 123/67; sats 96; AVPU alert	Acute Appendicitis PICU after theaters	No	314	Yes (64 min)	Yes 2.9 mMol/L (84 min)	Yes	Yes (204 min)	No	Yes (159 min)	No

a Manchester Triage flowchart, discriminator, and category;

b CRP value as measured at presentation;

* *This case is also included in Table 8*.

*In gray are the cases with PS6 interventions (that is: iv access, iv fluid bolus, iv antibiotics, and senior review) delivered within the first 60 min of arriving*.

*BP, blood pressure; CR, capillary refill; CRP, C-reactive protein; HR, heart rate; iv, intravenous; LRTI, lower respiratory tract infection; npa, nasopharyngeal aspirate; PED, pediatric emergency department; PICU, pediatric intensive care unit; PS6, pediatric sepsis 6 interventions; RR, respiratory rate; RSV, respiratory syncytial virus; T, temperature; sats, O2 saturations; SBI, serious bacterial infection*.

*The colours in the Triage code column represent the assigned triage urgency: red for ‘emergent’, orange for ‘very urgent’, yellow for ‘urgent’, and green for ‘standard’*.

**Table 7b:** Management of children who died in the PED (*n* = 1).

	**Age**	**Gender**	**Triage code^a^**	**Vital signs**	**Final diagnosis**	**IBI**	**CRP^b^ (mg/L)**	**PS6 interventions in the emergency department (time to intervention)**						
								**Iv access**	**Blood lactate (mMol/L)**	**Blood culture**	**Iv antibiotics**	**Iv fluid bolus**	**Senior review**	**Inotropes**
1	1 years	Male	Unwell child, Shock, emergent	In arrest	Meningococcal Septicaemia	Yes	25	Yes (2^*^ IO, at 31 min)	Yes 8.9 mMol/L (40 min)	Yes	Yes (31 min)	Yes (31 min)	Yes (0 min)	Yes

a Manchester Triage flowchart, discriminator, and category;

b *CRP value as measured at presentation*.

*CRP, C-reactive protein; IBI, invasive bacterial infection; Iv, intravenous; npa, nasopharyngeal aspirate; PS6, pediatric sepsis 6 interventions*.

*The colours in the Triage code column represent the assigned triage urgency: red for ‘emergent’, orange for ‘very urgent’, yellow for ‘urgent’, and green for ‘standard’*.

**Table 8:** Management of children (*n* = 4) on first presentation who were admitted to PICU or had IBI on revisit to PED.

	**Outcome on revisit**	**Time prior to revisit**	**Age**	**Gender**	**Triage code^a^**	**Vital signs**	**Initial working diagnosis**	**Diagnostics**	**Senior decision maker**	**Discharge plan initial visit**	**Final diagnosis**
1.1	PICU	32 h 42 m	2 years	Female	Vomiting, Signs of dehydration, urgent	HR 117; RR 32; T 36.5; CR 2 s or less; sats 98%; AVPU alert	*UTI*	Nil	No	Discharged home	Intussusception
1.2	PICU	21 h 38 m	2 years	Female	Urinary problems, Retention of urine, urgent	HR 143; RR 28; T 36.4; CR 2 s or less; Sats 100%; AVPU alert	*Gastroenteritis*	Urine MCS	No	Discharged home	Intussusception
2	PICU	41 h 26 m	1 month	Male	Worried parent, Recent problem, standard	HR 171; RR 80; T 37.9; Sats 98%; AVPU alert	*Bronchiolitis*	Nil	Yes	Admitted [discharged <24 h]	Bronchiolitis RSV+
3	IBI, admitted	35 h 32 m	4 years	Male	Unwell child, Hot child, very urgent	HR 152; RR 24; T39.2; CR 2 s or less; Sats 96%; AVPU alert	*Gastritis*	Nil	No	Discharge home	Pneumonia plus sepsis
4	RIP	8 h 0 m	1 years	Male	Unwell child, Recent problem, standard	HR 140; RR 50; T 39.0; CR 2 s or less; AVPU alert Repeated vital signs prior to discharge: 146 min; HR 142; RR 32; T 37; CR 2 s or less; Sats 100%; AVPU Alert	*Well child*	Nil	No	Discharged home	Meningococcal septicaemia

a *Manchester Triage flowchart, discriminator, and category*.

*BP, blood pressure; CR, capillary refill; HR, heart rate; IBI, invasive bacterial infection; PICU, pediatric intensive care unit; RR, respiratory rate; RSV, respiratory syncytial virus; T, temperature; sats, O2 saturations; urine MCS, urine microsopy culture and susceptibility; UTI, urinary tract infection*.

*The colours in the Triage code column represent the assigned triage urgency: red for ‘emergent’, orange for ‘very urgent’, yellow for ‘urgent’, and green for ‘standard’*.

An additional three children admitted to PICU (Appendix D) had fever and warning signs, but were excluded from final cohort. For one case most clinical data from the ED visit were missing, but review of microbiology yielded no positive bacteriology or virology. The other two children were excluded based on co-morbidity, one with status epilepticus and one with pneumonia.

In the total population, amongst children who were not eligible for inclusion in the final ICED cohort, there were an additional 21 PICU admissions (0.1%) and three deaths (0.01%) in the ED, with one more death in a child admitted to PICU via PED (Appendices D, E). Reasons for PICU admission were either a non-infectious illness (*n* = 14) or type I respiratory failure secondary to a respiratory tract infection without fever or hypothermia (*n* = 7), and not sepsis. These children mostly presented with high MTS urgency classification, and grossly abnormal vital signs; exceptions having a lower urgency classification included two children admitted to neonatal ITU for management of physiological neonatal jaundice, children with recurrence of seizures in PED, children with underlying comorbidity, clinical deterioration with reducing level of consciousness in diabetes, or worsening work of breathing in respiratory tract infections during stay in PED. Deaths were secondary to out of hospital cardiac arrest (*n* = 1), diabetic ketoacidosis (*n* = 1), cardiac arrest following major trauma (*n* = 1), and catastrophic injuries leading to death in PICU (*n* = 1), and not to sepsis.

### Management of Children With Confirmed Invasive Bacterial Infection

Six children (0.4%) had confirmed IBI with a bacterial pathogen identified in blood or CSF (Table 9). Amongst these is the child that died of meningococcal sepsis. The remaining five children were admitted to the pediatric ward; all but one were admitted after their first presentation to PED (Table 8). The one exception being a 4 year old child with pneumococcal septicaemia who was discharged from the PED <24 h before representing, with an initial working diagnosis of gastritis and without prescribing of antibiotics. This child was managed with iv antibiotics and admitted to the ward at second visit, and no serious complications occurred. The triage urgency as well as the number and expediency of PS6 interventions varied for children with IBI. None of these five children had signs of peripheral hypoperfusion at presentation, with reportedly normal capillary refill and unaltered mental state. Incidence of IBI in our cohort of febrile children with ≧1 warning signs was 0.39% (95% CI 0.29–0.49%), resulting in 256 children needing to be treated according to sepsis guideline recommendations for each child with IBI; for any serious outcome (*n* = 11), number needed to treat was 1:141.

**Table 9:** Management of children with invasive bacterial infection (*n* = 6).

	**Age**	**Gender**	**Triage code^a^**	**Vital signs**	**Initial working diagnosis**	**outcome**	**Comments**	**CRP^b^**	**PS6 interventions in the emergency department (time to intervention)**						
									**Iv access**	**Blood lactate (mMol/L)**	**Blood culture**	**Iv antibiotics**	**Iv fluid** **bolus**	**Senior** **review**	**Inotropes**
1	11 years	Female	Unwell child, Recent problem, standard	HR 137; RR 24; T 39.0; CR 2 s or less; Sats 99%; AVPU alert	Fever without source	Admitted	Focus: bacterial gastro-enteritis Blood culture and feces culture: *Salmonella paratyphi*	46 mg/L	Yes (176 min)	No	Yes	No (iv antibiotics started on ward)	No	Yes (136 min)	No
2	3 years	Female	Unwell child, Hot child, very urgent	HR 142 RR 26; T 39.5; CR 2 s or less; Sats 98%; AVPU alert	Septicaemia	Admitted	Focus: pneumonia, round consolidation on chest X Ray Leishmaniasis serology positive	37 mg/L	Yes (time unknown)	Yes 1.2 mMol/L (48 min)	Yes	Yes (40 min)	No	Yes (50 min)	No
3	2 months	Male	Unwell child, Hot child, very urgent	HR 165; RR 52; T 38; CR 2 s or less; Sats 100%; AVPU alert	Septicaemia	Admitted	Blood culture: *Haemophilus influenza*	37 mg/L	Yes (124 min)	Yes 2.5 mMol/L (84 min)	Yes	Yes (time unknown)	No	Yes (time unknown)	No
4	8 months	Female	Unwell child, Hot child, very urgent	HR 182; CR 2 s or less; Sats 100%; AVPU alert *2^*nd*^ set of obs (66 min)* HR 181; RR 30; T 38.0; Sats 100%; AVPU Alert	Septicaemia	Admitted	Blood and CSF culture: *Streptococcus pneumonia* CSF WBC: 816, polymorphs 73%	69 mg/L	Yes (208 min)	Yes 2.3 mMol/L (212 min)	Yes	Yes (279 min)	Yes (228 min)	Yes (121 min)	No
5	4 years	Male	Shortness of breath in children, Increased work of breathing, very urgent	HR 134; RR 38; T 38.0; CR 2 s or less; Sats 98%; AVPU alert	Chest infection	Admitted	Focus: pneumonia, bilateral consolidation on chest X ray Blood culture: *Streptococcus pneumoniae*	319 mg/L	Yes (72 min)	Yes 3.8 mMol/L (392 min)	Yes	Yes (72 min)	No	Yes (492 min)	No
6^*^	1 years	Male	Unwell child, Shock, emergent	In arrest	Meningococcal Septicaemia	RIP	Blood culture: *group B meningococcus*; meningococcal PCR positive	25 mg/L	Yes (2^*^ IO, at 31 min)	Yes 8.9 mMol/L (40 min)	Yes	Yes (31 min)	Yes (31 min)	Yes (0 min)	Yes

a Manchester Triage flowchart, discriminator, and category;

b CRP value as measured at presentation;

* *This case is also included in Tables 7b, 8*.

*BP, blood pressure; CSF, cerebrospinal fluid; CR, capillary refill; HR heart rate; IO, intra-osseus; Iv, intravenous;PS6, pediatric sepsis 6 interventions; RR, respiratory rate; T, temperature; sats O2, saturations*.

*The colours in the Triage code column represent the assigned triage urgency: red for ‘emergent’, orange for ‘very urgent’, yellow for ‘urgent’, and green for ‘standard’*.

## Discussion

### Principal Findings

This study describes the routine care of children with fever at increased risk of serious infections in a PED. A considerable proportion of febrile children presented with warning signs (41%). Serious infections were infrequent, and most children were managed conservatively without need for escalation of care and only limited use of resources.

Our cohort highlights the difficulties of introducing screening tools for sepsis in pediatric emergency care. In particular, any of the used thresholds for tachycardia and tachypnoea yielded considerable false positive results. Moreover, although hindered by missing values for blood pressure and WBC, the diagnostic performance of vital signs and sepsis criteria were insufficient (**Appendix C**). Lactate levels were not significantly associated with the decision to give iv fluid bolus or the presence of SBI or IBI/PICU.

Of the children with IBI or admitted to PICU, four children received all pediatric sepsis 6 interventions in the golden hour after arriving in PED, with expediency of PS6 interventions varying greatly in the others. As such, our data scope how the management of children with fever and warning signs in PED can be improved. Children with positive isolates in blood or CSF culture presented in a heterogeneous manner, with varying levels of triage urgency and severity of illness, consistent with findings of an earlier study (24), and showcasing the importance of careful clinical assessments of all febrile children with warning signs. For most of these children, expedited invasive interventions as recommended by the NICE sepsis guidelines were not merited in the clinical context. Our findings therefore align with the recent recommendations of the Surviving Sepsis group that advised on a 3 h window for observing and escalating care in children with warning signs of a serious infection but without evidence of shock (25).

### Comparison With Existing Literature

Early recognition of children with sepsis and subsequent timely management have proven vital for reducing sepsis related morbidity and mortality (4, 26–29). In both adults and children it was found that antibiotic treatment within the first hour of presentation influenced overall mortality (5, 30, 31). Similarly, adherence to clinical guidelines for the management for pediatric sepsis was associated with improved outcomes (8). However, a national audit amongst children with sepsis admitted to pediatric intensive care units in the UK showed poor adherence to the pediatric advanced life support guidelines, with non-compliance to guidelines in 62% of the children (28). Initial studies have shown that tools for managing childhood sepsis increased adherence to guidelines in PEDs in the United States and Australia (5, 32–36). However, it appears these studies selected more unwell children with clear signs of sepsis, and the impact on a non-selected population of febrile children presenting to PEDs remains unclear.

Previous studies showed that a considerable proportion of febrile children in PED fulfilled criteria for pediatric sepsis, and that most of these children were safely managed with a conservative observational clinical approach (12). Recently, based on the sepsis consensus-3 a bedside clinical score was proposed, the so called qSOFA (11, 37). A qSOFA score, modified for a pediatric population, validated well in a PICU population (21). However, our data showed that blood pressure was only recorded in 7%, and was not routinely performed at the time of triage. Measuring blood pressure routinely in children at the time of triage comes with a number of practical difficulties in the PED.

The role of tachycardia as appropriate indicator for serious infections in secondary emergency care has been debated. One systematic review found limited diagnostic value of heart rate in detecting serious infections (38). Another review proposed altering normal values for both heart rate and respiratory rate (20). The new NICE sepsis guidelines used these updated thresholds, but our data didn't show an immediate clinical benefit. Similarly, one study found no advantage of using temperature dependent normal values of heart rate for detecting sepsis over APLS threshold values (39). On the other hand, temperature dependent threshold values for respiratory rate performed better in children with lower respiratory tract infections than APLS threshold values (40). Furthermore, several studies discussed limitations of the interrater variability and interpretation of a prolonged capillary refill, often used in clinical sepsis scores (41, 42). One study did not find an association between peripherally or centrally measured capillary refill and serious infections in the ED (43), contrasting with the often perceived usefulness of capillary refill as a reliable clinical sign of peripheral perfusion in the context of sepsis in high incidence clinical environments. Equally, altered mental state is a potential indicator of poor cerebral perfusion and this has been adopted as a warning sign in sepsis scores and guidelines. In children, however, it might be more difficult to detect early, more subtle, changes in mental state, such as irritability or lethargy, which can often also be explained by other contributing factors. In our cohort, only few children had prolonged capillary refill or an altered mental state; five out of six children with IBI had normal capillary refill (2 s or less) and unchanged mental state.

Finally, lactate levels play a central role in guiding resuscitation and administration of intravenous fluid bolus in sepsis (44–49). There is limited evidence base for this approach in pediatric emergency care (50). Our association between lactate levels, intravenous fluid bolus, and hospitalization is likely self-perpetuating. Our data also suggest that more clinical parameters are being taken into account, other than lactate level alone, when deciding on the need for intravenous fluid bolus: several children with high lactate levels did not receive a fluid bolus. Known confounding factors, such as use of bronchodilators or capillary sampling methods, need to be taken into consideration. Moreover, guidelines from the European Pediatric Advanced Life Support (EPALS) do not support the use of fluid in children without clinical evidence of septic shock (51). In addition, in children with suspected myocardial dysfunction fluids should be titrated particularly carefully, and congenital heart disease in young children and myocarditis can mimic sepsis presentations. Similarly, the recent recommendations by the Surviving Sepsis campaign did not identify a lactate threshold to suggest fluid bolus in children with sepsis, and urged mindful administration of fluids in the absence of intensive care facilities (25).

### Clinical Implications and Future Research

Combined, the reported use of resources and expediency of interventions can be used as a surrogate to reflect the low overall severity of illness of the majority of children with fever and warning signs seen in PEDs. Our data provide an estimate of the required sensitivity and specificity of potential sepsis tools to improve current clinical pediatric emergency care. The introduction of tools for the early detection of sepsis might correctly improve recognition and time to interventions in some children. However, this will need to be positioned against the burden of false positives in a resource stretched environment, the hazards of overtreating and overdiagnosis of potentially serious conditions which aren't so, and the dilution of capacity of a department to deal with the true positive owing to the larger number of relatively well children. This is much in contrast to the hypothesized benefits of recognizing children with sepsis early, potentially resulting in improved mortality rates, shorter PICU admissions, and fewer requirements for interventions at a later stage of the admission. In particular, the risks of fluid overload in critically ill children are well recognized (51–54). Let alone risks of anaphylaxis and other adverse reactions from antibiotics, or extravasation injuries from intravenous access.

The senior decision maker plays a pivotal role in all this, as outlined in the NICE sepsis algorithms that state that any child triggering the sepsis pathway should be seen within an hour by a senior decision maker, defined as a pediatric or emergency care registrar ST3 or above. This senior decision maker has the ability to de-escalate care at any time, as was shown in a recent implementation study (55). It is unclear if senior decision makers would have influenced the potentially delayed management of the four children with IBI or PICU admission with previous ED visit (0.3%, Table 8).

More research on largescale datasets will be needed to validate the NICE sepsis guidelines and to better define early predictors of sepsis. Combinations of clinical predictors and biomarkers might improve the risk assessment and likelihood of children having sepsis at first assessment. Many biomarkers have been proposed for diagnosing sepsis, with best evidence available C-reactive protein and Procalcitonin (56). Procalcitonin appears to perform better in young infants and neonates, as well as in children with a short duration of fever (57), reflecting the shorter inflammatory response time of Procalcitonin compared to that of C-reactive protein (58). However, no biomarker is capable for diagnosing sepsis or SBI in isolation (59, 60). In our study, initial levels of C-reactive protein ranged widely for those with IBI or admitted to PICU. Also, extensive health economic analyses on the impact of sepsis tools in pediatric emergency care are yet to be undertaken. Finally, a review of all children admitted to PICU (Appendix D) showed that several children with infections did not present with fever, but other signs such as respiratory distress. Future studies should consider broader eligibility criteria.

### Strengths and Limitations

This study is the first to describe a prospective cohort of febrile children targeted by the new NICE sepsis guidelines for improving the early detection of pediatric sepsis. All clinical data were collected in a standardized manner as part of routine clinical care, in a period preceding the release of these guidelines. Another strength is the near complete data available for eligible children, mostly as a result of introducing the study protocol within routine clinical care. Moreover, all medical staff were specialized in pediatric emergency care, including being certified in advanced pediatric life support ensuring high quality of collected data.

As a limitation, our data only reflect practice in a single center, and multicentre studies across multiple sites and countries are needed. At the time of data collection, other than local guidelines, no stringent guidance for following a pediatric sepsis protocol nor a sepsis trigger system were in place, partly explaining the low compliance with the PS6 care bundle. We didn't perform additional qualitative research to better understand compliance with the PS6 care bundle or the role of the senior decision maker, and this will need further research. We didn't collect biochemical data to show the incidence of children fulfilling definitions for severe sepsis; but we expect this to be very low in our population considering the low incidence of true sepsis and PICU admissions. Also, we did not evaluate the change in practice following the introduction of the NICE sepsis guideline (13). We included data from a 9-month period, meaning seasonal influence cannot be ruled out. Furthermore, the clinical warning signs for pediatric sepsis are liable to interrater variability. However, introducing tools with clinical signs and symptoms inherently mean this needs to be taken into account to ensure generalisability. We only considered data from initial triage, and not from any clinical deterioration in the emergency department. Finally, we looked solely in children with no significant prior medical history. Our population consisted for only a small proportion of children with significant co-morbidities (<5%). Children with a complex medical history are generally at increased risk of serious infections and more susceptible for complications of serious infections (61). The disease course and the signs and symptoms of serious infections can differ from otherwise healthy children, and they do not necessarily mount a fever. The validity of clinical warning signs and symptoms, and the role of sepsis screening tools, in children with a complex medical history will need to be studied in future studies.

## Conclusion

Many febrile children have warning signs of possible sepsis on arrival to PED, and few requiring management for true sepsis. Introducing pathways for early escalation of care for children fulfilling criteria of suspected sepsis might have unwanted effects on the overall management of febrile children in the emergency department.

## Data Availability Statement

The raw data supporting the conclusions of this article will be made available by the authors, without undue reservation.

## Ethics Statement

The studies involving human participants were reviewed and approved by Imperial College London. Written informed consent from the participants' legal guardian/next of kin was not required to participate in this study in accordance with the national legislation and the institutional requirements. Written informed consent was obtained from the minor(s)' legal guardian/next of kin for the publication of any potentially identifiable images or data included in this article.

## Author Contributions

RN was guarantor of this paper, attests that all listed authors meet authorship criteria and that no others meeting the criteria have been omitted, wrote the first draft of the manuscript, and was responsible for data analysis. RN, RJ, ML, JH, and IM were all responsible for designing this study and its implementation. JH, IM, and ML were responsible for obtaining funding. RN and RJ were mainly responsible for data collection. All authors had full access to the data, have contributed significantly to the writing of this manuscript, and have read the final version of this manuscript they have approved the manuscript as submitted.

## Conflict of Interest

The authors declare that the research was conducted in the absence of any commercial or financial relationships that could be construed as a potential conflict of interest.

## Abbreviations

BP: blood pressure
CI: confidence interval
CR: capillary refill
CSF: cerebrospinal fluid
GCS: Glasgow coma scale
HR: heart rate
IBI: invasive bacterial infection
IO: intra-osseous
IM: intramuscular
IV: intravenous
LR: likelihood ratio
LRTI: lower respiratory tract infection
MTS: Manchester Triage System
NPV: negative predictive value
Npa: nasopharyngeal aspirate
PED: pediatric emergency department
PICU: pediatric intensive care unit
PPV: positive predictive value
PS6: pediatric sepsis 6
qSOFA: quick Sequential Organ Assessment Failure
RR: respiratory rate
RSV: respiratory syncytial virus
SBI: serious bacterial infections
SIRS: Systemic Inflammatory Response Syndrome
WBC: white blood cell count.
