# Supplementary material for: Management of Children With Fever at Risk for Pediatric Sepsis: A Prospective Study in Pediatric Emergency Care
Source: Front Pediatr. 2020 Sep 17;8:548154. doi: 10.3389/fped.2020.548154 (PMC7527403; doi:10.3389/fped.2020.548154)
Supplement: Supplementary file 1 [file Table_1.DOCX]

## Appendix A. Sepsis definitions and sepsis scoring systems

Refs:

Schlapbach et al, Intensive Care Medicine, 2018 (22)

Goldstein et al, Ped Crit Care Med, 2005 (11)

| 1. **Systemic Inflammatory Response Syndrome (SIRS)**   ***Positive if 2 or more are present*** | | | |  |
| --- | --- | --- | --- | --- |
|  |  |  |  |  |
| **Age Group** | **Heart Rate** | **Respiratory Rate** | **Leukocyte Count** | **Temperature** |
| **1 month - <2 years** | <90 or >180 | >34 | >17.5 or <5 | <36 or >38.5 |
| **2 to <5 yrs** | >140 | >22 | >15.5 or <6 | <36 or >38.5 |
| **>=5 to 12 yrs** | >130 | >18 | >13.5 or <4.5 | <36 or >38.5 |
| **>=12 to <16 yrs** | >110 | >14 | >11 or <4.5 | <36 or >38.5 |

| 1. **Pediatric age-adapted quick SOFA (qSOFA)**   ***Positive if score is 2 or above***  ***Note:***  ***This should prompt calculation of a SOFA score as per Seps-3 task force***  ***Surviving Sepsis Campaign 2017: qSOFA used to identifyatients a thigh risk of mortality, not to diagnose sepsis.*** | | | |
| --- | --- | --- | --- |
|  |  |  |  |
| **Parameter** |  |  |  |
|  |  | **Score** |  |
| ***Tachypnea*** | Definition | 0 | 1 |
| Respiratory Rate | <2 years | <=34 | >34 |
| (breaths per minute) | 2 to 5 yrs | <=22 | >22 |
|  | >5 to 12 yrs | <=18 | >18 |
|  | >12 to <18 yrs | <=14 | >14 |
|  |  |  |  |
| ***Altered mentation*** |  |  |  |
| AVPU: if Voice, Pain, Unresponsive 1 point | | | |
| *Schlapbach et al used GCS for model: (not used in this paper)* | | | |
| *Glasgow coma scale* | Definition | 0 | 1 |
| GCS main model | GCS_15 | 15 | <15 |
| GCS 13 model | GCS_13 | 13-15 | <13 |
| GCS 14 model | GCS_14 | 14-15 | <14 |
|  |  |  |  |
| ***Hypotension*** |  |  |  |
|  | Definition | 0 | 1 |
| Systolic blood pressure | 1 month - <2 years | ≥75 | <75 |
| (mmHg) | >=2 to < 5 yrs | ≥74 | <74 |
|  | >=5 to <12 yrs | ≥83 | <83 |
|  | >=12 to <16 yrs | ≥90 | <90 |

1. **Sepsis TRUST UK criteria:**

***Positive if at least 2 are present***

| **Sepsis TRUST UK criteria:** | **Parameter coded as:** |
| --- | --- |
| Core temperature <36.0 OR >38.5 degrees Celsius | - Axillary temperature ( <36.0 OR >38.5 degrees Celsius |
| Inappropriate tachycardia (APLS thresholds) | - Age <= 1 yr: heart rate >160 beats per minute - Age 1 - <=2 yrs: heart rate >150 beats per minute - Age 2 - <=5 yrs: heart rate >140 beats per minute - Age 5 - <= 112 yrs: heart rate > 120 beats per minute - Age >12 yrs: heart rate >100 beats per minute |
| Altered mental state (including: sleepiness, irritability, lethargy, floppiness) | - AVPU: Voice, Pain, Unresponsive, - or NICE fever warning signs positive for: abnormal level of activity, abnormal response to social cues, decreased level of consciousness, decreased rousability |
| Reduced peripheral perfusion, or prolonged capillary refill | - Capillary refill time >2 seconds |
