# Supplementary material for: Management of Children With Fever at Risk for Pediatric Sepsis: A Prospective Study in Pediatric Emergency Care
Source: Front Pediatr. 2020 Sep 17;8:548154. doi: 10.3389/fped.2020.548154 (PMC7527403; doi:10.3389/fped.2020.548154)
Supplement: Supplementary file 2 [file Table_2.DOCX]

## Appendix B. Different thresholds for defining tachycardia and tachypnoea in all children with fever (n=5,156)

|  | *Thresholds for respiratory rate for tachypnoea,*  *categorised for age groups (in breaths per minute)* | | | | *Thresholds for heart rate for tachycardia,*  *categorised for age groups (in beats per minute)* | | | |
| --- | --- | --- | --- | --- | --- | --- | --- | --- |
| Age groups | Tachypnoea, APLS defined | N (%) | Tachypnoea, NICE sepsis thresholds | N (%) | Tachycardia, APLS defined | N (%) | Tachycardia, NICE sepsis thresholds | N (%) |
| 1 mo – <1 year | Aged < 2 months: >60  Aged 2 – < 12 months: >40 | 250/757 (33%) | AMBER: 50 - 59  RED: >= 60 | AMBER: 66 (9%)  RED: 46 (6%) | >160 | 216/768 (28%) | AMBER: 150 – 159  RED: >= 160 | AMBER: 154 (20%)  RED: 230 (30%) |
| 1 – <2 year | >35 | 332/949 (35%) | AMBER: 40 - 49  RED: >= 50 | AMBER: 148 (16%)  RED: 66 (7%) | >150 | 452/980 (46%) | AMBER: 140 – 149  RED: >= 150 | AMBER: 151 (15%)  RED: 468 (48%) |
| 2 – <5 years | >30 | 488/1,607 (30%) | Aged 2 - <3 years:  AMBER: 40 - 49  RED: >= 50  Aged 3 - <5 years:  AMBER: 35 - 39  RED: >= 40 | AMBER: 138 (9%)  RED: 99 (6%) | >140 | 615/1,647 (37%) | Aged 2 - <3 years:  AMBER: 140 - 149  RED: >= 150  Aged 3 - <5 years:  AMBER: 130 - 139  RED: >= 140 | AMBER: 305 (19%)  RED: 541 (33%) |
| 5 – <16 years | Aged 5 - <12 years: >25  Aged >= 12 years: >20 | 536/1,417 (38%) | Aged 5 - <6 years  AMBER: 24 - 28  RED: >= 29  Aged 6 - <8 years  AMBER: 24- 26  RED:>= 27  Aged 8 - <12 years  AMBER: 22 - 24  RED: >= 25  Aged >= 12 years  AMBER: 21 - 24  RED: >= 25 | AMBER: 660 (47%)  RED: 289 (20%) | Aged 5 - <12 years: >120  Aged >= 12 years: >100 | 624/1,484 (42%) | Aged 5 - <6 years  AMBER: 120 - 129  RED: >= 130  Aged 6 - <8 years  AMBER: 110 - 119  RED: >= 120  Aged 8 - <12 years  AMBER: 105 - 114  RED: >= 115  Aged >= 12 years  AMBER: 91 - 130  RED: >130 | AMBER: 397 (27%)  RED: 523 (35%) |
|  | **Overall:** | **1,606/4,730 (34%)** | AMBER:  RED:  **ANY** | 1,012 (21%)  500 (11%)  **1,512 (32%)** | **Overall:** | **1,907/4,879 (39%)** | AMBER:  RED:  **ANY** | 1,007 (21%)  1,762 (36%)  **2,769 (57%)** |
