# Supplementary material for: Management of Children With Fever at Risk for Pediatric Sepsis: A Prospective Study in Pediatric Emergency Care
Source: Front Pediatr. 2020 Sep 17;8:548154. doi: 10.3389/fped.2020.548154 (PMC7527403; doi:10.3389/fped.2020.548154)
Supplement: Supplementary file 3 [file Table_3.DOCX]

## Appendix C. Diagnostic performance of trigger and scoring systems for sepsis.

| **SBI**  **(n=111)** |  | Sensitivity  (95% CI) | Specificity  (95% CI) | PPV  (95% CI) | NPV  (95% CI) | LR +  (95% CI) | LR –  (95% CI) |
| --- | --- | --- | --- | --- | --- | --- | --- |
|  | APLS tachycardia | 0.76 (0.67 – 0.84) | 0.25 (0.23 – 0.28) | 0.07 (0.06 – 0.09) | 0.93 (0.90 – 0.95) | 1.01 (0.91 – 1.13) | 0.95 (0.68 – 1.33) |
|  | NICE RR AMBER^1^ | 0.33 (0.23 – 0.43) | 0.68 (0.66 – 0.71) | 0.06 (0.05 – 0.10) | 0.93 (0.91 – 0.95) | 1.04 (0.76 – 1.44) | 0.98 (0.84 – 1.14) |
|  | NICE RR RED^1^ | 0.32 (0.22 – 0.44) | 0.78 (0.75 – 0.80) | 0.10 (0.07 – 0.15) | 0.93 (0.91 – 0.95) | 1.45 (1.04 – 2.04) | 0.87 (0.75 – 1.01) |
|  | APLS tachypnea | 0.56 (0.47 – 0.66) | 0.45 (0.43 – 0.48) | 0.08 (0.06 – 0.10) | 0.93 (0.91 – 0.95) | 1.03 (0.87 – 1.22) | 0.96 (0.78 – 1.20) |
|  | NICE HR AMBER^1^ | 0.65 (0.46 – 0.80) | 0.44 (0.39 – 0.49) | 0.08 (0.05 – 0.12) | 0.94 (0.90 – 0.97) | 1.15 (0.89 – 1.50) | 0.80 (0.51 – 1.27) |
|  | NICE HR RED^1^ | 0.86 (0.77 – 0.92) | 0.17 (0.15 – 0.20) | 0.07 (0.06 – 0.09) | 0.94 (0.90 – 0.97) | 1.04 (0.95 – 1.14) | 0.80 (0.47 – 1.36) |
|  | SIRS positive | 0.73 (0.64 – 0.81) | 0.47 (0.44 – 0.49) | 0.09 (0.07 – 0.12) | 0.96 (0.94 – 0.97) | 1.37 (1.21 – 1.55) | 0.58 (0.43 – 0.79) |
|  | qSOFA positive | 0 (0 – 0.05) | 0.01 (0.00 – 0.01) | 0 (0 – 0.00) | 1 (1 – 1.00) | na^3^ | na^3^ |
|  | Sepsis Trust positive | 0.45 (0.41 – 0.50) | 0.65 (0.62 – 0.67) | 0.08 (0.06 – 0.11) | 0.93 (0.92 – 0.95) | 1.15 (0.91 – 1.45) | 0.92 (0.79 – 1.07) |
|  |  |  |  |  |  |  |  |
|  | NICE RR AMBER^1^ | 0.43 (0.12 – 0.80) | 0.68 (0.66 – 0.71) | 0.01 (0.00 – 0.02) | 1.00 (0.99 – 1.00) | 1.36 (0.57 – 3.21) | 0.84 (0.44 – 1.59) |
|  | NICE RR RED^1^ | 0.43 (0.12 – 0.80) | 0.77 (0.75 – 0.80) | 0.01 (0.00 – 0.04) | 1.00 (0.99 – 1.00) | 1.89 (0.80 – 4.47) | 0.74 (0.39 – 1.41) |
| **IBI and PICU**  **(n=11)** | APLS tachypnea | 0.40 (0.14 – 0.73) | 0.45 (0.42 – 0.48) | 0.00 (0.00 – 0.01) | 0.99 (0.98 – 1.00) | 0.73 (0.34 – 1.55) | 1.33 (0.80 – 2.22) |
|  | APLS tachycardia | 0.91 (0.57 – 1.00) | 0.25 (0.23 – 0.27) | 0.01 (0.00 – 0.02) | 1.00 (0.98 – 1.00) | 1.21 (1.01 – 1.47) | 0.36 (0.06 – 2.35) |
|  | NICE HR AMBER^1^ | 1.00 (0.05 – 1.00) | 0.43 (0.39 – 0.48) | 0.00 (0.00 – 0.02) | 1.00 (0.98 – 1.00) | 1.77 (1.64 – 1.91) | na^2^ |
|  | NICE HR RED^1^ | 1.00 (0.65 – 1.00) | 0.17 (0.15 – 0.19) | 0.01 (0.00 – 0.02) | 1.00 (0.99 – 1.00) | 1.21 (1.18 – 1.24) | na^2^ |
|  | SIRS positive | 0.02 (0.01 – 0.03) | 1.00 (1.00 – 1.00) | 0.08 (0.05 – 0.12) | 0.99 (0.99 – 0.99) | 7.97 (4.88 – 13.03) | 0.99 (0.98 – 0.99) |
|  | qSOFA positive | 0.18 (0.03 – 0.52) | 1.00 (0.99 – 1.00) | 0.25 (0.04 – 0.64) | 0.99 (0.99 – 1.00) | 46.67 (10.56 – 206.3) | 0.82 (0.62 – 1.08) |
|  | Sepsis Trust positive | 0.64 (0.32 – 0.88) | 0.65 (0.62 – 0.67) | 0.01 (0.01 – 0.03) | 1.00 (0.99 – 1.00) | 1.79 (1.14 – 2.82) | 0.56 (0.26 – 1.23) |

Based on number of disease episodes (n=1,551), with vital signs used as measured at first presentation to predict for final outcomes of SBI, and IBI – PICU.

Appendix A for definitions of SIRS, qSOFA and Sepsis Trust; any one NICE RED or any two NICE AMBER should trigger escalation of care and senior clinician review; SIRS, qSOFA and Sepsis Trust criteria considered positive if 2 or more items present. Any missing item of SIRS, qSOFA and Sepsis Trust equals 0 for total score.

^1^ vs no AMBER or RED

^2^ na, not applicable; all cases with IBI – PICU had NICE AMBER (n=1) or RED (n=10) at first presentation.

^3^ Only 8 cases had enough data to calculate qSOFA score >1, mostly due to large % of missings for BP and low number of children with reduced level of consciousness; no cases of SBI had qSOFA score >1.

CI Confidence interval; HR heart rate; RR respiratory ratel PPV positive predictive value; NPV negative predictive value; LR + positive likelihood ratio; LR – negative likelihood ratio; SBI Serious bacterial infection; IBI Invasive bacterial infection; PICU; Paediatric intensive care unit admission.
