# Supplementary material for: Management of Children With Fever at Risk for Pediatric Sepsis: A Prospective Study in Pediatric Emergency Care
Source: Front Pediatr. 2020 Sep 17;8:548154. doi: 10.3389/fped.2020.548154 (PMC7527403; doi:10.3389/fped.2020.548154)
Supplement: Supplementary file 4 [file Table_4.DOCX]

## Appendix D. PICU admissions (n=29 in the total cohort of n=18,104 visits)

| **age** | **gender** | **Triage code^** | **Vital signs** | **PED working diagnosis** | **comments** | **In ICED cohort*** | **RIP** |
| --- | --- | --- | --- | --- | --- | --- | --- |
| 10.49 | male | Unwell child, Airway compromised, emergent | BP 112/64; HR 70; AVPU pain; 100% in high flow; CR 2 seconds or less unresponsive  *2^nd^ set of obs*  GCS 6; BP 101/50; sats 100% in 15L | Hypoglycaemia in pt with diabetes |  | NOT ELIGIBLE, no fever | no |
| .94 | female | Shortness of breath in children, Inadequate breathing, emergent | *No obs on arrival available*  *1^st^ set of obs*  Sats 65%; Nil else | Lower respiratory tract infection | Metapneumovirus isolated from npa  Comorbidity: T21 | NOT ELIGIBLE, no fever | no |
| 2.48 | male | Fits, Currently fitting, emergent | *Fitting on arrival*  *1^st^ set of obs*  HR 130;BP 156/124;RR 28; Sats 100% (in 15L) | Status epilepticus | Comorbidity: Dravets syndrome | NOT ELIGIBLE, no fever | no |
| 11.25 | female | Shortness of breath in children, Inadequate breathing, emergent | *No obs on arrival available*  *1^st^ set of obs*  T37.3; HR 160; RR 32; CR 2s; AVPU Alert; Sats 90% | asthma |  | NOT ELIGIBLE, no fever | no |
| 13.12 | male | Shortness of breath in children, Inadequate breathing, emergent | HR 132; RR 44; AVPU Voice; Sats 88%; CR 2 - <4 s; T 37.5 | asthma |  | NOT ELIGIBLE, no fever | no |
| .17 | male | Unwell child, Unresponsive, emergent | *No obs on arrival available*  Unresponsive and apnea on arrival  *1^st^ set of obs*  HR 185; T 36.7; RR 58; Sats 100 % (in 10L); CR 2 seconds or less; AVPU Alert | Apnea, bronchiolitis | RSV isolated from npa | NOT ELIGIBLE, no fever | no |
| 5.20 | male | Fits, Airway compromised, emergent | *fitting on arrival*  *1^st^ set of obs*  HR 129; RR 24; BP 115/85; CR 2 seconds or lesss; AVPU Alert/voice; Sats 100%; | Status epilepticus | Comorbidity: known seizure disorder | NOT ELIGIBLE, no fever | no |
| 2.11 | female | Fits, Currently fitting, emergent | Fitting on arrival; GCS 9  *1^st^ set of obs*  T 36.4; HR 148; BP 113/72; RR 40; Sats 100% (in 15L); CR 2 seconds or less; AVPU Pain  *15 minutes after 1st obs*  GCS 3; T 36.7; HR 136;  RR intubated | Status epilepticus | Influenza A isolated from npa | NOT ELIGIBLE, no fever | no |
| 3.07 | male | Major trauma, Airway compromised, emergent | [trauma notes] | Pulmonary contusion and pneumothorax |  | NOT ELIGIBLE, no fever | no |
| 13.75 | female | Major trauma, airway compromised,  emergent | [trauma notes] | Splenic laceration and pulmonary contusion | Angiography; Embolization + | NOT ELIGIBLE, no fever | no |
| 14.32 |  | Major trauma, Inadequate breathing,  emergent | [trauma notes] | Aorctic dissection |  | NOT ELIGIBLE, no fever | YES |
| 5.86 | male | Major trauma, Inadequate breathing,  emergent | [trauma notes] | Major trauma, Retinal detachment; Left distal femoral fracture | PICU, then theatres and ward | NOT ELIGIBLE, no fever | no |
| 2.73 | Female | Shortness of breath in children, Increased work of breathing, very urgent | HR 163; RR 52;T 37.7; CR 2 seconds or less; sats 88; AVPU alert | Lower respiratory tract infection | Adenovirus isolated from npa | NOT ELIGIBLE, no fever | no |
| *.26* | *male* | *Unwell child,*  *Responding to voice or pain only,*  *Very urgent* | *T 37; Sats 98%; RR 38; HR 139; AVPU alert* | *Other* | *To* ***NICU****;*  Comorbidity: *prematurity at gestational age 25 weeks*  *Managed with CPAP* | *NOT ELIGIBLE, no fever* | *no* |
| 12.95 | female | Abdominal Pain in Children, Signs of severe pain, very urgent | HR 139; BP 108/81; RR 28; CR 4 seconds or more | Diabetic ketoacidosis |  | NOT ELIGIBLE, no fever | no |
| 1.32 | male | Shortness of breath in children, Increased work of breathing,  Very urgent | Sats 95%; RR 64; HR 142; AVPU alert; CR<2s | Severe viral induced wheeze | Iv aminophylline and MgSO4  High dependency level care | NOT ELIGIBLE, no fever | no |
| 1.71 | male | Shortness of breath in children, Increased work of breathing, very urgent | T 36.5; Sats 100  RR 44; HR 189; AVPU alert | Wheeze / chest infection | Rhinovirus isolated in from npa  High dependency level care | NOT ELIGIBLE, no fever | no |
| 11.16 | female | Shortness of breath,  Inappropriate history,  urgent | Sats 98%; RR 28; HR 120; AVPU alert; CR<2s | Status epilepticus | Comorbidity: cerebral palsy | NOT ELIGIBLE, no fever | no |
| 7.22 | male | Shortness of breath in children, Low SaO2, urgent | T 36.5; Sats 82%; RR 52; HR 150; AVPU alert; CR<2s | Chest infection and wheeze |  | NOT ELIGIBLE, no fever | no |
| *.01* | *male* | *Unwell child, Not feeding, urgent* | *T 37; Sats 100; RR 33; HR 117* | *jaundice* | ***NICU*** *for 5 days*  *managed with phototherapy* | *NOT ELIGIBLE*, no fever | *no* |
| *.02* | *Female* | *Unwell child,*  *Not feeding,*  *urgent* | *T 36.5, Sats 100%, RR 24, HR 103, AVPU alert, CR<2s* | *Jaundice* | ***NICU*** *for 7 days*  *bilirubin above exchange threshold, managed with phototherapy; hypernatriemia* | *NOT ELIGIBLE* no fever | *no* |
| 13.47 | female | Unwell child, , urgent | HR 169;RR 36;T 38.6; CR 2 seconds or less; Sats 95% AVPU alert | Pneumonia | Comorbidity: muscular dystrophia on home BiPAP | EXCLUDED,  Comorbidity | no |
| 15.06 | male | Collapsed Adult, Airway compromised, emergent | Nil available (no notes traceable) | Sepsis | No clinical notes available  Travelling from Kuwait  No pathogen identified (full Ix done) | EXCLUDED,  missing data and language barrier | no |
| 3.92 | female | Unwell child, Hot child, very urgent | CR 4 seconds or more; AVPU alert | Status Epilepticus | Comorbidity: recurrent (febrile) seizures | EXCLUDED, comorbidity | no |
| 11.26 | female | Unwell child, Hot child, very urgent | HR 136; RR 32; T 40.5; Sats 98%; AVPU voice | Encephalitis | Influenza A isolated from npa | **YES** | no |
| 1.92 | female | Fits, Airway compromised, emergent | HR 185; RR 30; T 39; CR >2 - <4 seconds; Sats 100%; AVPU unresponsive | Status Epilepticus | RSV and influenza isolated from npa | **YES** | no |
| 2.46 | female | Abdominal Pain in Children, Signs of severe pain, very urgent | HR160; RR26; T 37.9; CR 2 seconds or less; BP 94/63; Sats 100%; AVPU alert | Intussusception | PICU after theatres | **YES** | no |
| .09 | male | Shortness of breath in children, Inceased work of breathing, very urgent | HR 170; RR 110; T 38.3; Sats 88%; AVPU pain | Lower respiratory tract infection | human metapneumovirus isolated from npa | **YES** | no |
| 10.95 | male | Abdominal Pain in Children, Signs of moderate pain, urgent | HR 160; RR 22; T 40; BP 123/67; sats 96%; AVPU alert | Acute Appendicitis | PICU after theatres | **YES** | no |

^ Manchester Triage flowchart, discriminator, and category

BP: blood pressure; CR capillary refill; GCS Glasgow coma scale; HR heart rate; NICU neonatal intensive care unit; Npa: nasopharyngeal aspirate; PICU paediatric intensive care unit; RR respiratory rate; T temperature; sats O2 saturations

^*^ if YES, then duplicated case in table 8a; cases in italics (n=3) were admitted to NICU
