# Supplementary material for: Management of Children With Fever at Risk for Pediatric Sepsis: A Prospective Study in Pediatric Emergency Care
Source: Front Pediatr. 2020 Sep 17;8:548154. doi: 10.3389/fped.2020.548154 (PMC7527403; doi:10.3389/fped.2020.548154)
Supplement: Supplementary file 5 [file Table_5.DOCX]

## Appendix E. Deaths in PED (n=4 in the total cohort of n=18,104 visits)

|  | **age** | **gender** | **Triage code^** | **Vital signs** | **PED Working diagnosis** | **comments** | **In ICED cohort** |
| --- | --- | --- | --- | --- | --- | --- | --- |
| 1 | 1.27 | Male | Unwell child, Shock, emergent | In arrest | Meningococcal Septicaemia | Revisit same day | YES |
| 2 | 13.61 | Female | Unwell child, Unresponsive, emergent | HR 119; CR 2 seconds or less; Sats 73%; AVPU unresponsive | Diabetic Ketoacidosis |  | NOT ELIGIBLE |
| 3 | .74 | Female | Unwell child, Airway compromised, emergent | In arrest | Other, cardiac arrest on arrival |  | NOT ELIGIBLE |
| 4 | 6.53 | Female | Major trauma, Airway compromised, emergent | In arrest | Other, cardiac arrest with major trauma |  | NOT ELIGIBLE |

^ Manchester Triage flowchart, discriminator, and category; AVPU level of consciousness CR capillary refill; HR heart rate; sats oxygen saturations
