# Supplementary material for: Management of Children With Fever at Risk for Pediatric Sepsis: A Prospective Study in Pediatric Emergency Care
Source: Front Pediatr. 2020 Sep 17;8:548154. doi: 10.3389/fped.2020.548154 (PMC7527403; doi:10.3389/fped.2020.548154)
Supplement: Supplementary file 6 [file Data_Sheet_2.DOC]

STROBE Statement—Checklist of items that should be included in reports of ***cohort studies***

|  | Item No | Recommendation |
| --- | --- | --- |
| **Title and abstract** | 1 | (*a*) Indicate the study’s design with a commonly used term in the title or the abstract  **Title, p1** |
|  |  | (*b*) Provide in the abstract an informative and balanced summary of what was done and what was found  **Abstract, p3** |
| Introduction | | |
| Background/rationale | 2 | Explain the scientific background and rationale for the investigation being reported  **Introduction, p4** |
| Objectives | 3 | State specific objectives, including any prespecified hypotheses  **Introduction, final paragraph, p5** |
| Methods | | |
| Study design | 4 | Present key elements of study design early in the paper  **Methods, section: design, setting and participants, p5** |
| Setting | 5 | Describe the setting, locations, and relevant dates, including periods of recruitment, exposure, follow-up, and data collection  **Methods, section: design, setting and participant, p5**  **Methods, section: outcome measures, p7**  **Methods, section: data collection, p9** |
| Participants | 6 | (*a*) Give the eligibility criteria, and the sources and methods of selection of participants. Describe methods of follow-up  **Methods, section: design, setting and participant, p5**  **Methods, section: outcome measures, p7** |
|  |  | (*b*) For matched studies, give matching criteria and number of exposed and unexposed  ***n/a*** |
| Variables | 7 | Clearly define all outcomes, exposures, predictors, potential confounders, and effect modifiers. Give diagnostic criteria, if applicable  **Methods, section: outcome measures, p7**  **Methods, section: data collection, p9** |
| Data sources/ measurement | 8* | For each variable of interest, give sources of data and details of methods of assessment (measurement). Describe comparability of assessment methods if there is more than one group  **Methods, section: design, setting and participant, p5**  **Methods, section: outcome measures, p7**  **Methods, section: data collection, p9** |
| Bias | 9 | Describe any efforts to address potential sources of bias  *For our study limitations are addressed in the discussion section; for the Methods section no specific confounding variables were considered relevant for reporting measures.* |
| Study size | 10 | Explain how the study size was arrived at  *No formal power calculation for this study as not believed relevant to outcome measures and reporting measures*. |
| Quantitative variables | 11 | Explain how quantitative variables were handled in the analyses. If applicable, describe which groupings were chosen and why  **Methods, statistical analyses, p9** |
| Statistical methods | 12 | (*a*) Describe all statistical methods, including those used to control for confounding  **Methods, statistical analyses, p9**  No advanced statistical analyses were used for this study |
|  |  | (*b*) Describe any methods used to examine subgroups and interactions  *n/a* |
|  |  | (*c*) Explain how missing data were addressed  *n/a; only complete cases/available data were analysed for the purposes of this study* |
|  |  | (*d*) If applicable, explain how loss to follow-up was addressed  *n/a* |
|  |  | (*e*) Describe any sensitivity analyses  *n/a* |
| Results | | |
| Participants | 13* | (a) Report numbers of individuals at each stage of study—eg numbers potentially eligible, examined for eligibility, confirmed eligible, included in the study, completing follow-up, and analysed  **Figure 1**  **Results, Study population, p10** |
|  |  | (b) Give reasons for non-participation at each stage  **Figure 1** |
|  |  | (c) Consider use of a flow diagram  **Figure 1** |
| Descriptive data | 14* | (a) Give characteristics of study participants (eg demographic, clinical, social) and information on exposures and potential confounders  **Table 2, 3** |
|  |  | (b) Indicate number of participants with missing data for each variable of interest  **Table 2,3,4; Appendix B** |
|  |  | (c) Summarise follow-up time (eg, average and total amount)  *n/a* |
| Outcome data | 15* | Report numbers of outcome events or summary measures over time  **Table 3**  **Results, vital signs and sepsis criteria, p10**  **Results, Management of children at risk for serious infection, p11**  **Results, Management of children admitted to PICU, p12**  **Results, Management of children with confirmed invasive bacterial infections, p13**  **Appendix C** |
| Main results | 16 | (*a*) Give unadjusted estimates and, if applicable, confounder-adjusted estimates and their precision (eg, 95% confidence interval). Make clear which confounders were adjusted for and why they were included  **Results, Management of children at risk for serious infection, p11**  **Figure 3**  *No regression or stratified analysis were performed.* |
|  |  | (*b*) Report category boundaries when continuous variables were categorized  **Appendix A, B** |
|  |  | (*c*) If relevant, consider translating estimates of relative risk into absolute risk for a meaningful time period  *n/a* |
| Other analyses | 17 | Report other analyses done—eg analyses of subgroups and interactions, and sensitivity analyses  **Appendix C: diagnostic performance of trigger and scoring systems for sepsis** |
| Discussion | | |
| Key results | 18 | Summarise key results with reference to study objectives  **Discussion, Principal findings, p14** |
| Limitations | 19 | Discuss limitations of the study, taking into account sources of potential bias or imprecision. Discuss both direction and magnitude of any potential bias  **Discussion, strengths and limitation, p19** |
| Interpretation | 20 | Give a cautious overall interpretation of results considering objectives, limitations, multiplicity of analyses, results from similar studies, and other relevant evidence  **Discussion, Comparison with existing literature, p15**  **Discussion, Clinical implications and future research, p17** |
| Generalisability | 21 | Discuss the generalisability (external validity) of the study results  **Discussion, Comparison with existing literature, p15**  **Discussion, Clinical implications and future research, p17**  **Discussion, strengths and limitation, p19** |
| Other information | | |
| Funding | 22 | Give the source of funding and the role of the funders for the present study and, if applicable, for the original study on which the present article is based  **Funding, p20** |

*Give information separately for exposed and unexposed groups.

**Note:** An Explanation and Elaboration article discusses each checklist item and gives methodological background and published examples of transparent reporting. The STROBE checklist is best used in conjunction with this article (freely available on the Web sites of PLoS Medicine at http://www.plosmedicine.org/, Annals of Internal Medicine at http://www.annals.org/, and Epidemiology at http://www.epidem.com/). Information on the STROBE Initiative is available at http://www.strobe-statement.org.
